# Plant Resin Delivery by Nanovectors as an Emerging Approach to Boost Solubility, Permeability and Bioavailability

**DOI:** 10.3390/pharmaceutics17010053

**Published:** 2025-01-03

**Authors:** Eleonora Truzzi, Giulia Vanti, Lucia Grifoni, Eleonora Maretti, Eliana Leo, Anna Rita Bilia

**Affiliations:** 1 Department of Life Sciences, University of Modena and Reggio Emilia, Via G. Campi 103, 41125 Modena, Italy; eleonora.truzzi@unimore.it (E.T.); eleonora.maretti@unimore.it (E.M.); eliana.leo@unimore.it (E.L.); 2 Department of Chemistry “Ugo Schiff” (DICUS), University of Florence, Via Ugo Schiff 6, 50019 Sesto Fiorentino, Italy; giulia.vanti@unifi.it (G.V.); lucia.grifoni@unifi.it (L.G.)

**Keywords:** plant resins and their essential oils, *Boswellia* and *Commiphora*, *Pistacia*, *Pinus* and *Picea*, *Ferula*, *Dracaena*, *Copaifera*, nanovectors, active or nanocarrier-forming constituents, enhanced stability and bioavailability

## Abstract

Resins are complex mixtures of natural constituents containing non-volatile and volatile terpenes, in combination with gums and polyphenols, used since ancient times for their medicinal properties. Current research has evidenced their therapeutic value with a plethora of activities. The main limits of resins and their constituents for their clinical use are low water solubility, poor stability and bioavailability. Therefore, nanovectors including vesicles, solid lipid nanoparticles, micelles, nanoemulsions, microemulsions and mesoporic nanoparticles have been investigated to optimize the biopharmaceutical properties after topical or oral administration of resins or fractions from them, including essential oils or single constituents. In this review, we report the results evidencing that developed nanovectors were able to entrap high amounts of resins or their components, modify the release properties, enhance their cellular uptake and penetration across biological barriers and optimize the biopharmaceutical properties. In addition, the resins or their fractions as enhancer penetration molecules can optimize the architecture and properties of nanovectors in their capacity to circumvent biological barriers. Although no clinical studies have been reported until now, nanovectors represent a huge platform for upgrading therapies and emerging new treatments of resins such as wound healing therapy.

## 1. Introduction

Plant resins are complex blends of secondary constituents, mainly characterized by nonvolatile terpenoids (di- or triterpenes; rarely both) in combination with other constituents, mainly represented by essential oils (mono- and sesquiterpenes, denominated oleoresins), gums (polyterpenes, denominated gum resins), oil and gum (named oleogum resins) and polyphenols (phenylpropanoids, lignans, flavonoids, denominated balsams). Very frequently, oleogum resins are pleasantly fragrant, owing to the presence of the essential or volatile oil in some quantity [1].

Resins are synthesized in specified subcellular structures and can be produced spontaneously or due to stress (wound, pathogen attack). Their physiological role is probably to protect the plant from insects, fungi or other infections, or to close wounds. Resins principally constituted of terpenoids are internally formed, while resins containing polyphenols are produced on the surface. Due to this specific composition, resins are generally insoluble in water but soluble in alcohol, oil, chloroform, and ether [2]. Resins and resinous extracts of plant origin have been regarded as an important plant resource in traditional medicine and represent the oldest plant-based products, used by humans for thousands of years in various applications, principally for their antimicrobial, anti-inflammatory, antispasmodic, analgesic, and digestive properties. Their effects and applications in traditional medicine are widely described in Egyptian, Roman, Greek, Chinese and Oriental literature and include frankincense, myrrh, Dragon’s blood and ferulae resins, among the most widely used [3,4,5].

In the early 20th century, resins were still used in therapy; indeed, “Mastisol”, used for wound healing, was developed using mastic, styrax, methyl salicylate and alcohol [6]. The Merck’s Annual Report in 1913 referred to Mastisol as a resinous preparation containing 5% of mastic (terebinth 15 g, mastic 12 g, resin (any) 25 g, resin alb. 8.0 g, alcohol 90% 180 g) recommended as a wound dressing [7]. In the same period, Dr. Norman Moore of the Royal College of Physicians suggested another formulation based on resins: 20 g mastic, 50 g benzol, 20 drops linseed oil, 10 g colophony and 7 g Venice turpentine [8]. Finally, the formulation is also reported in Medicamenta, an Italian theoretical-practical guide for medical doctors and pharmacists [9]. Indeed, resins and their fractions, including EOs and isolated constituents, due to their lipid nature, are generally insoluble in water and petroleum ether, but soluble in alcohol, chloroform, and ether, and are quite soluble in acetone and fixed and volatile oils [4,5]. Their hydrophobic nature represents a major issue when resins are used as medicinal products because their low solubility limits their pharmacokinetics and biodistribution, representing a major challenge in the field of drug delivery. This limit can be overcome by their formulation in nanoparticles, improving their efficacy, specificity and targeting ability, and possibly obtaining a controlled and sustained drug release. In addition, nanovectors protect their cargo from degradation in the biological media, improve bioavailability, and optimize the cellular uptake [10,11]. The aim of this review was to give an overview on the published data on nanovectors loaded with resins or their fractions or isolated constituents.

## 2. Nanocarriers Are Smart Delivery Systems in Nanomedicine

Nanocarriers have shown great promise as drug deliverers and have been extensively investigated in the past few decades as transport modules for drugs. These colloidal drug carrier systems typically have submicron particle sizes, generally from a few to 250 nm [10], but many studies describe larger particles (up to 500 nm in diameter) [11]. Size, shape, surface charge, and surface chemistry have a fundamental role in the optimization of intracellular delivery of drugs and the pharmacokinetic and pharmacodynamics parameters of the drugs [10]. Commonly used nanocarriers include organic (natural or synthetic) and inorganic materials, or even hybrid nanodelivery systems. Different types of nanomaterials can be used to formulate nanocarriers, which are capable of being loaded with hydrophobic and/or hydrophilic drugs [11]. The inclusion of the drug in the nanocarriers has many advantages in terms of biopharmaceutical properties with respect to the naked drug. Indeed, nanoencapsulation of the drug enhances drug stability and solubility, with a consequent enhanced absorption, and decreases elimination and metabolism of drugs, resulting in an increased bioavailability and reduced therapeutic dose and toxicity. Other advantages of nanodrug delivery systems could be targeted drug delivery and drug-controlled release, minimizing systemic side effects compared to free drugs [10,11].

**Figure 1:**
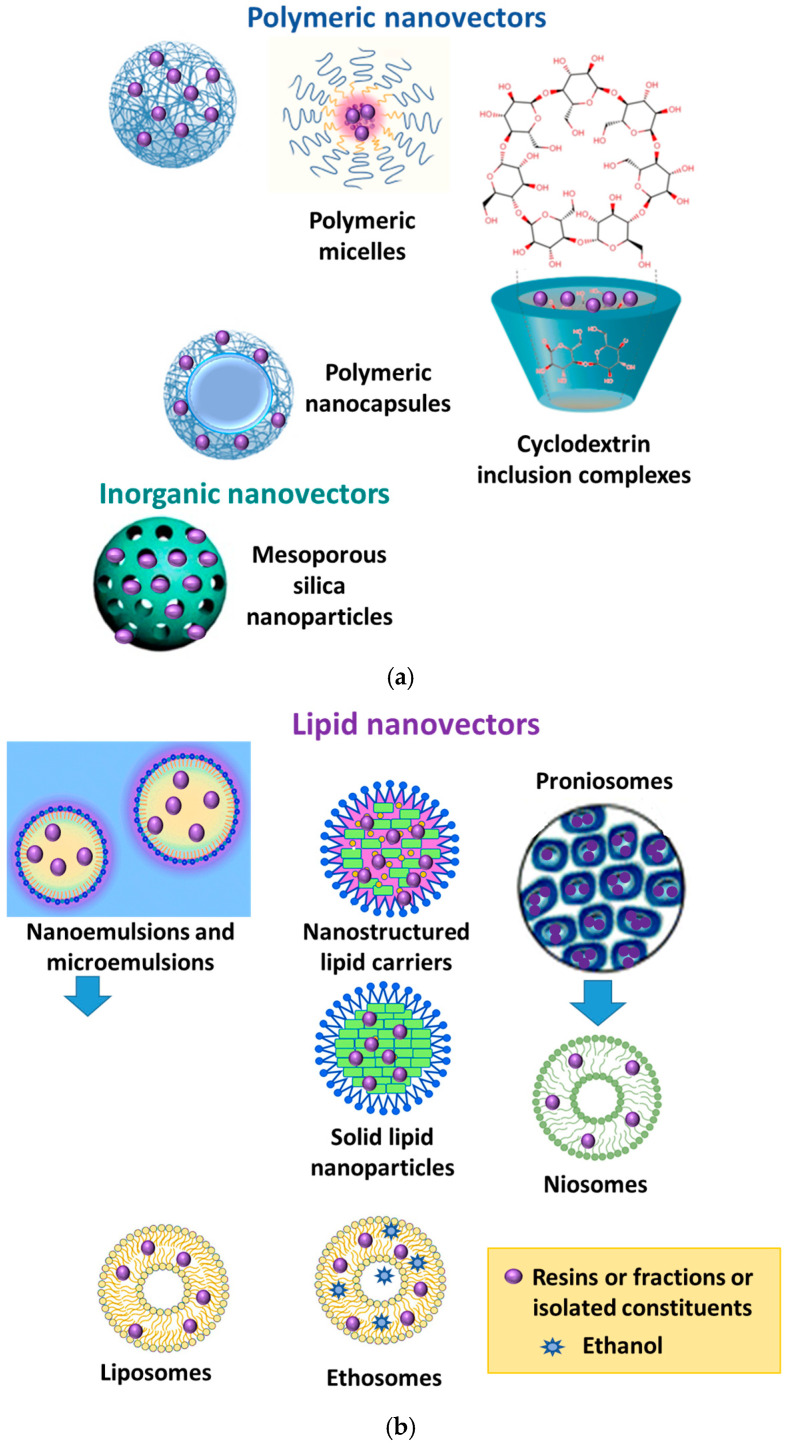
Nanovectors developed for the delivery of plant resins: (**a**) polymer-based systems, inorganic systems and hybrid systems; (**b**) lipid-based systems.

Classification of the nanocarriers is mainly based on the nature of the nanocarriers and includes polymer-based systems, lipid-based systems, inorganic systems and hybrid systems (Figure 1). Polymeric nanocarriers are composed of natural, semisynthetic or synthetic polymers (Figure 1). Their selection is established by the characteristics of the drugs to be encapsulated and by their performances in terms of biodegradability and biocompatibility [12]. Natural and synthetic polymers have been used for the formulations of resin-based nanoparticles and nanocapsules (Figure 1). Polymeric micelles have also been used as resin-loaded nanovectors. They consist of block copolymers made of hydrophobic and hydrophilic units, which can spontaneously associate in an aqueous solution, originating the micelle at a certain concentration (critical micellar concentration, CMC) [13] (Figure 1).

Lipid-based nanocarriers are widely used for the loading of resins, their fractions or isolated constituents. They include vesicles, nanoparticles and nanoemulsions (Figure 1b). Nanoemulsions and microemulsions are both nanosized emulsions, constituted of an oil phase and an aqueous phase [14]. Microemulsions are biphasic, isotropic, homogeneous and thermodynamically stable. Nanemulsions are thermodynamically unstable, even they show long-term stability when compared to macroemulsions [15]. In some cases, nanoemulsions and microemulsions have a low viscosity, leading to little retention time and spreadability, which can be overcome by developing nanoemulgel and microemulgel using a suitable gelling agent. There are many advantages of the nanoemulgel with respect to nanoemulsion and microemulsion, principally extraordinary drug loading capacity, improved viscosity, retention time and spreadability, little skin irritation and enhanced thermodynamic stability [16]. These microemulsions are very interesting as nanovectors for EO because they can decrease their volatility and increase both the water solubility and stability of EO in the presence of oxygen, light and humidity [17].

Vesiscles are also used to load resins, fractions of resins or single constituents. They are characterized by a bilayer structure spontaneously formed by natural or synthetic phospholipids or non-ionic surfactants, respectively denominated by liposomes and niosomes (Figure 1). They are very versatile nanocarriers because they can encapsulate both hydrophilic and hydrophobic drugs, respectively, in the aqueous compartment and the lipophilic bilayer. The principal advantage of these nanocarriers is their tremendous biocompatibility and safety [18]. Cholesterol can be replaced by other natural constituents such as saponins, which can impart to the bilayer a characteristic architecture, resulting in enhanced drug loading and drug skin penetration [19]. These nanovescicles can be also formulated in thermosensitive gels, which are of particular interest because the gel is formed in situ due to a sol–gel phase transition near body temperature, optimizing the prolonged release of the loaded drugs [20]. Finally, proniosomes are pre-vesicular formulations which can overcome the limitations of niosomes and general vesicular systems. The gel texture of proniosomes can be converted into the niosomes immediately upon hydration, simply in situ by absorbing water from the skin [21].

Lipid nanoparticles (Figure 1) have a lipid core consisting of solid or solid-plus-liquid lipids, and include natural and synthetic components and surfactants, mainly represented by polysorbates, phospholipids and bile salts. Lipid nanoparticles possess great biocompatibility, extraordinary biodegradability, and high safety, and they are more stable than vesicles. Another advantage is the easy and cheap industrial upscaling [22]. To limit the extrusion phenomena and optimize the entrapment efficiency of drugs, the blend of liquid and solid lipids at room temperature is strongly recommended to form the matrix [23].

Since their isolation, cyclodextrins (CDs) have been recognized for their applications in drug delivery [24,25]. Nanosponges based on CDs can be produced by reacting the CD unit with an appropriate crosslinking agent. These sponges have an astonishing ability to encapsulate drugs due to the three-dimensional networks with spherical shape [26].

Among the inorganic particles, mesoporous bioactive glass nanoparticles (MPGs) represent a smart class of nanoparticles and have been used for loading extracts from resins. Indeed, bioactive glasses (BGs) have been known for 50 years, made of SiO_2_–Na_2_O–CaO–P_2_O_5_ systems, greatly recognized as new biomaterials for regenerating bone. MPGs have high biocompatibility and quick bioactive response, representing useful nanoparticles for regenerative medicine [27,28].

## 3. Plant Resins Loaded in Nanocarriers

### 3.1. Frankincense

Frankincense, called also olibanum, is a natural oleogum resin, which is obtained by a deep longitudinal cut in the trunk of the *Boswellia* trees, a milky juice exudate that, if exposed to air, forms a yellowish-brown oleogum resin. Frankincense, possessing a variety of pharmacological effects, has been used since ancient times for cosmetic and medicinal purposes. Four species of *Boswellia are generally used to produce frankincense*, *principally B. serrata* Roxb. and *B. sacra* Flueck. (*syn. B. carteri* Birdw.), *in addition to B. papyrifera *(Del.) Hochst and *B. frereana* Birdw., which are located in Northeast Africa (Somalia), India, the Middle East, China and East Africa, respectively [29]. Frankincense is mainly composed of diterpenes and triterpenes (30–60%), which are soluble in alcohol. The most characteristic constituents are the pentacyclic triterpenes, denominated boswellic acids; particularly, the most diffused are α- and β-boswellic acids, acetylated α- and β-boswellic acids and two very active constituents, 11-keto-β-boswellic acid (KBA) and 3-O-acetyl-11-keto-β-boswellic acid (AKBA). Indeed, boswellic acids are widely studied for their anti-inflammatory and immunomodulatory activities, being KBA and AKBA, the most potent constituents in down-regulating the production of cytokines and inhibiting the enzymes accountable for inflammation. Nowadays, some products based on the boswellic acid fraction of this resin are available on the market [30]. The EO (5–10%) is also a component of frankincense, obtained by hydro-distillation, whose main constituents are monoterpene hydrocarbons and oxygenated monoterpenes. It is reported that EO has antiseptic and astringent properties, and it is widely marketed [31]. The remaining constituents are represented by water-soluble gum, principally pentose and hexose saccharides [32].

**Figure 2:**
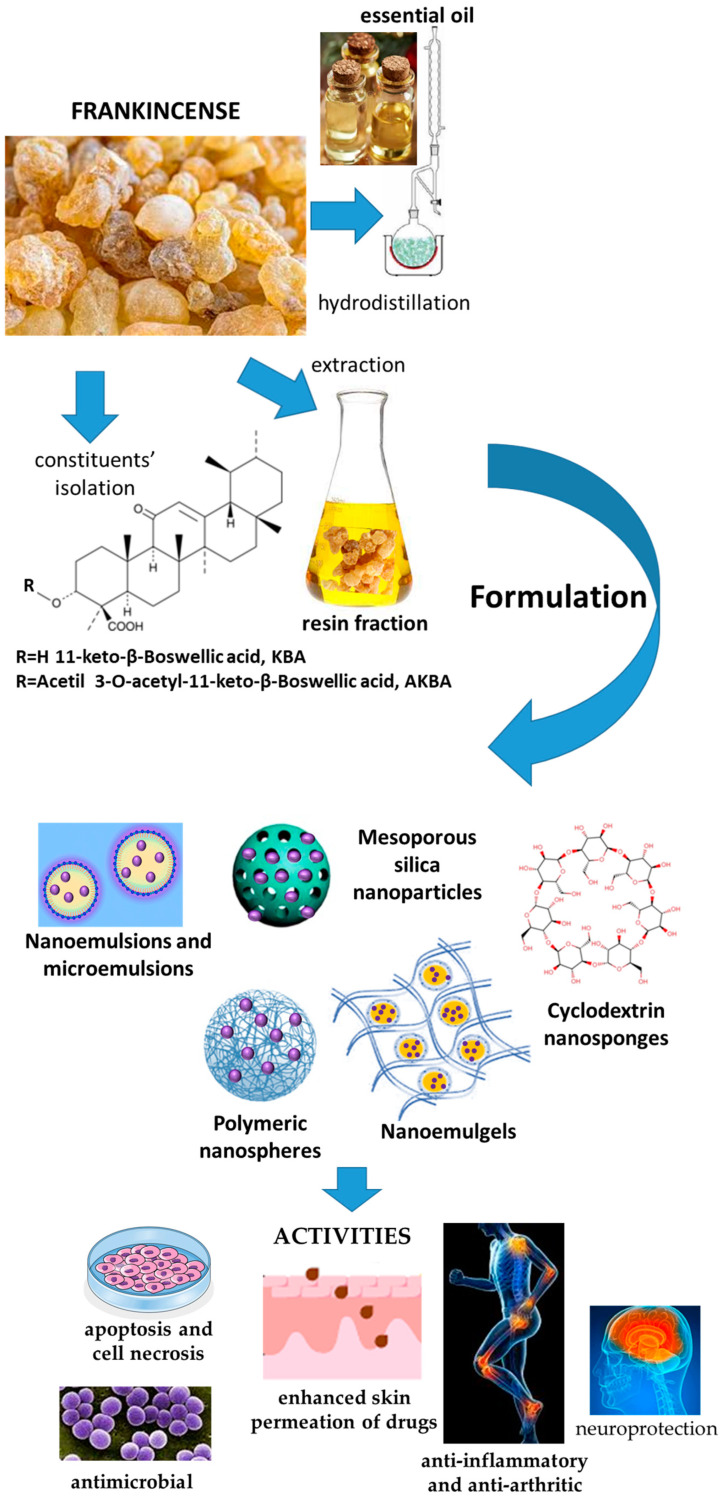
Nanovectors developed for the delivery of frankincense and main effects.

However, in vivo anti-inflammatory activity of frankincense and isolated boswellic acids in both animals and human volunteers after oral administration is very limited even at high doses (3 g/d). This is due to the poor oral bioavailability due to the high lipophilicity and scarce water solubility, in addition to a rapid phase-1 metabolism and poor intestinal permeability, with an elimination half-life of 4.5 ± 0.55 h, classifying the resin and boswellic acids as a BCS class IV compounds. The hampered boswellic acid transport across the intestine also includes instability in the gastro-intestinal tract, intestinal metabolism by CYP3A4 and accumulation within the cells and mucus [33,34,35,36,37]. Numerous studies have evidenced that frankincense formulated in nanocarriers can improve both bioavailability and efficacy. A summary of the nanoformulations and proven effects is reported in Figure 2.

A recent study investigated the six major boswellic acids (β-boswellic acid, 3-acetyl-β-boswellic acid, 11-keto-β-boswellic acid, 3-acetyl-11-keto-β-boswellic acid, β-boswellic alcohol, and 3-acetyl-11-hydroxy-β-boswellic acid) from frankincense to investigate their molecular properties and determine water solubility, partition coefficient (Log P), gastrointestinal stability, adsorption–desorption kinetics, and permeability studies. All the tested molecules were found to be unstable in simulated gastrointestinal fluids and intestinal S9 fractions. Their apparent permeability was in the range of 0.52 ± 0.05 × 10^−6^ to 2.84 ± 0.14 × 10^−6^ cm/s, confirming their accumulation (35–55%) inside the enterocytes [38]. A few studies have also investigated topical administration of frankincense using conventional formulations. Generally, the local effects can be obtained, but at the same, due to high lipophilicity (log P = 8) of boswellic acids, this alternative route shows a low biovalaibility [38], with molecules with intermediate lipophilicity (log P = 2–3) being the best to permeate via both the lipid and polar micro environments within the intercellular route [39]. To overcome these drawbacks, nanoformulations could really represent strategies to enhance permeability on topical application and to optimize the bioavailability after oral administration. In truth, there are many studies reporting the use of surfactant micelles to enhance the solubility of boswellic acids within the micelle core, but these were not considered in this review because these kinds of micelles are not true vectors. Generally, polysorbates are used as emulsifiers, and most of these preparations are sold as food supplements [40].

The first study reported in the literature on a nanoformulation of frankincense is the development of a polymeric nanomicelle containing a single main constituent from frankincense, AKBA, for topical use as an anti-inflammatory and anti-arthritic formulation. The nanomicelles were produced by a radical polymerization of *N*-isopropylacrylamide, vinylpyrrolidone and acrylic acid, and had a size of approximately 45 nm while drug loading was 45%. The results of release studies of AKBA from nanomicelles in an aqueous buffer (pH 7.4) were 23 and 55% after 2 and 8 h, respectively, demonstrating a sustained release. Ex vivo skin permeation studies using excised abdominal skin were carried out using a Carbomer 940 gel of AKBA and AKBA-loaded nanomicelles gelled with Carbopol 940, containing the same amount of AKBA. A three-fold increase in skin permeability of the gel containing the micelles was found when compared with AKBA gel, containing the same amount of AKBA. Finally, the AKBA polymeric nanomicelle gel showed significantly enhanced anti-inflammatory and anti-arthritic activity compared with AKBA gel [41].

In a further study, AKBA was loaded in nanoparticles made of poly lactic-co-glycolic acid (PLGA) to improve its oral bioavailability and anti-inflammatory activity in rats in a paw edema model induced by carrageenan. The mean size of the developed nanoparticles loaded with AKBA was 179.6 nm, with a polydispersity index of 0.276. Entrapment efficiency was 82.5%. Nanoparticles loaded with AKBA had an increased anti-inflammatory activity compared with unformulated AKBA in in vivo studies. The bioavailability of AKBA-loaded nanoparticles evidenced a higher peak plasma concentration of about six times when compared with unformulated AKBA. Moreover, the t_1/2_ and AUC of AKBA were also enhanced by two- and nine-fold, respectively, in AKBA-NPs when compared with unformulated AKBA [42].

A petroleum ether fraction of frankincense was formulated in a microemulsion and tested for transdermal administration and anti-inflammatory properties. The fraction included different constituents, namely KBA, AKBA, 3-acetyl-β-boswellic acid, β-boswellic acid, α-boswellic acid, 15-methyl-4-hexdecenoic acid, sterols and fatty acids. For the microemulsion, capmul MCM C8 was selected as the oil phase, the surfactants were Tween 80 and Labrasol and PEG 400 was selected as the co-surfactant. The weight ratios of surfactants and co-surfactant (Smix) were 1:1, 2:1 and 3:1. According to the pseudo ternary phase, two microemulsions were selected, composed of Tween 80: PEG 400 at 1:1 and 2:1 ratios, with oil contents of 7.78 and 17.5%, respectively. Particle sizes were about 67 nm and 90 nm, respectively; polydisperisity was 0.05 for both micromulsions; and drug encapsulation was 83 and 100%, respectively.

The two microemulsions were evaluated for ex vivo skin permeation through excised rat skin, and the drug permeation from both was sustained for 24 h, resulting in approximately 97 and 30%, respectively. Both microemulsions were safe without irritant effects. The in vivo anti-inflammatory activity was evaluated using a carrageenean-induced rat paw edema model. The first microemulsion had an inhibition of about 50%, while the second displayed an inhibition of 35% (*p* < 0.05) [43].

PLGA nanoparticles containing trehalose as cryoprotectant and polyvinyl acetate as surfactant were developed and loaded with KBA. The particle size was about 152.6 nm with a polydispersity index of 0.194, the drug loading was 13.28%, and the entrapment efficiency was 79.7%. The release of KBA from nanoparticles was biphasic, with an initial burst release followed by a sustained release phase, reaching 61.5% at 72 h. Antiinflammatory activity was investigated at 50 mg/kg p.o. dose in Sprague–Dawley rats having carrageenan-induced paw edema. The nanoparticles showed 60.8% inhibition of rat paw edema after 5 h, while that of unformulated KBA was 34.9% after the same time (1.7-fold increase of anti-inflammatory activity). An oral bioavailability study investigated with the same dose and route of administration demonstrated a seven-fold increase when compared to unformulated KBA [44].

Moreover, AKBA was loaded in carboxymethyl chitosan nanoparticles with a size of 132 ± 18 nm, polydispersity indexes of 0.112 ± 0.094, and a zeta potential of +24.5 ± 3.1 mV. AKBA drug entrapment efficiency was 87.5 ± 6.5%, while drug loading was 32.5 ± 5.0%.

The in vitro release of nanoparticles loaded with AKBA showed a burst drug release (60%) during the first 6 h. Then, the rate gradually reduced, and the cumulative release of AKBA was over 70% within 96 h.

Pharmacokinetic studies were carried out in male Sprague–Dawley rats aged 8–10 weeks, and the performance of the AKBA in the nanoparticles was compared with that of unformulated AKBA. The plasma AUC of AKBA loaded in nanoparticles (373.6 ± 65.9 mg min/mL) was 3.7-fold higher than that of the unformulated AKBA (100.2 ± 21.4 mg min/mL); the t_1/2_ (274.3 ± 68.9), MRT_inf_ (373.6 ± 65.9), and V_ss_ (291.7 ± 58.2) of AKBA loaded in nanoparticles were increased by 5.7-, 1.65-, and 3.5-fold, respectively, while Cl was decreased from 99.8 ± 11.0 to 26.8 ± 3.2 mL/min/kg compared with the unformulated AKBA. After 24 h, AKBA was still detected in the plasma in the group treated with the nanoparticles, while in the group treated with the unformulated AKBA, after 12 h, it was no longer present in the blood. Differences in the tissue’s distribution were also found 2 and 6 h after the administration of nanoparticle or unformulated AKBA. AKBA loaded in nanoparticles was mainly distributed to the blood and liver, while after administration of unformulated AKBA, it was concentrated in the kidney and spleen, due to its phagocytation by the reticuloendothelial system. AKBA concentration in the brain after the administration of the nanoparticles was about three times that of the unformulated treated AKBA group (*p* < 0.05). Comparative studies on the effect of the unformulated AKBA were carried out in primary neurons with an oxygen–glucose deprivation (OGD) model and, in animals, with the middle cerebral artery occlusion (MCAO) model. Cell viability in the group treated with nanoparticles was higher (70 ± 5%) than the OGD group (36 ± 3%) and the unformulated AKBA group (54 ± 6%) (*p* < 0.05). The studies in MCAO animals evidenced that the nanoparticles noticeably reduced cerebral infarct volume by approximately 32% and improved the neural function deficit (*p <* 0.05) [45].

A proniosomal gel made of Span 40, lecithin and cholesterol was prepared and optimized by experimental design. Frankincense containing not less than 65% of boswellic acids was loaded in the formulation. The final concentration of frankincense was 1%, entrapment efficiency was 98.52%, and particle size was 707.9 nm. An in vitro skin permeation study evidenced a sustained release, reaching 84.83 ± 0.153 mg/cm^2^ in 24 h. The formulation was evaluated for the anti-inflammatory activity in the carrageenan-induced hind paw edema method using Wistar rats. Rats were treated with the proniosomal gel containing 1% of frankincense and a commercial conventional gel of diclofenac (1%). The inhibition of paw edema 5 h after treatment resulted in 42.5 ± 0.024% and 50 ± 0.02%, respectively, with proniosomal gel (1%) and diclofenac gel (1%), respectively, compared to the control. Proniosomal gel at the doses of 1%, 2% and 3% of frankincense after single application demonstrated a dose-dependent significant difference (*p* < 0.001) in paw edema when compared with the control animals [46]. 

A natural dressing for skin disease was obtained by loading frankincense in two nanoemulsions and then dispersed in natural hydropolymer bacterial nanocellulose (BNC). The O/W nanoemulsions contained Imwitor 375 (glyceryl citrate/lactate/linoleate/oleate), glyceryl monooleate, *Helianthus annuus* seed oil, propylene glycol and water. The W/O/W nanoemulsions contained Imwitor 375 (glyceryl citrate/lactate/linoleate/oleate), ethyl oleate, propylene glycol and water. Both nanoemulsions were loaded with 1% frankincense. The mean particle size of the empty O/W and W/O/W nanoemulsions were about 149 and 113 nm, respectively. The loading of frankincense did not influence sizes (only an increase of 1–4 nm). Polydispersity was less than 0.16, and the zeta potentials of the empty nanoemulsions were –72 mV and –80 mV, respectively. The loading of frankincense did not change the surface charge. The dynamic viscosity of both loaded nanoemulsions was measured with a rheometer at a shear rate of 100/s and a temperature of 20 °C. It resulted 6.67 ± 0.17 mPa*s and 3.92 ± 0.17 mPa*s, respectively. pH values were 5.68 (O/W) and 5.56 (W/O/W). Good storage stability over 30 days at temperatures between 4 and 32 °C was found. Nanoemulsions were loaded into the BNC without changing its characteristics (water absorption and retention, softness, and pressure stability in a relevant way). In addition, the sterilization of the formulations by an electron-beam procedure was proved. The biocompatibility of final formulations was checked ex ovo by a shell-less hen’s egg test. Finally, tape stripping experiments using porcine skin evidenced a dependency of the frankincense penetration into skin. The formulation containing the W/O/W nanoemulsion facilitated drug transport into deeper skin layers [47].

An ethanol fraction of frankincense was loaded in amino-functionalized mesoporous bioactive glass nanoparticles having a composition of 58% SiO_2_, 37% CaO and 5% P_2_O_5_ and prepared by sol–gel-modified co-precipitation. After the preparation, the surfaces of the nanoparticles were modified by using 3-aminopropyltriethoxysilane. The surface area of nanoparticles without functionalization was about 100 m^2^/g with pores of 2 nm, while the functionalized nanoparticle surface area was about 111 m^2^/g with pores of 6 nm. The zeta potential was −21.7 mV for non-functionalized nanoparticles and −11.8 mV, becoming more negative after the addition of frankincense (−32.2 mV). Frankincense dissolved in methanol (10% *w*/*v*) was added to the functionalized nanoparticles, stirred for 24 h, and dried at room temperature. The loading capacity of functionalized nanoparticles was higher (9.9 ± 0.1 mg) than that of nanoparticles without functionalization (1.9 ± 0.2 mg) per 500 mg nanoparticles. In the release studies, in the case of functionalized nanoparticles loaded with frankincense, the released frankincense was about 52% in 6 h, followed by a constant and slower release up to 168 h. The cumulative release from functionalized nanoparticles was 99.9% of the total loading after 144 h, while the non-functionalized nanoparticles showed a release of 96.6% of frankincense within 24 h. Cell culture studies using human osteoblastic-like cells (MG-63) indicated better cell viability of the nanoparticles loaded with frankincense when compared to the unloaded nanoparticles. The antibacterial properties of both functionalized nanoparticles and frankincense-loaded functionalized nanoparticles were evaluated by the zone-of-inhibition formation. Frankincense-loaded functionalized nanoparticles were significantly active against *S. aureus* (Gram-positive) and a slight reduction of Gram-negative (*E. coli*) bacterial colonies was also found [48].

An ethyl acetate fraction of frankincense was loaded in nanosponges prepared with hydroxypropyl-β-cyclodextrin (HPβ-CD) and diphenyl carbonate (cross-linker) in the ratio (1:5, 3:1, 4:1 and 5:1), obtaining spherical and uniformly sized nanosponges. The nanocarriers were loaded with dexamethasone sodium sat or the ethyl acetone fraction of frankincense. Four formulations containing dexamethasone (D1–D4) had entrapment efficiencies between 98.52 ± 0.07 and 99.64 ± 1.40%. Additionally, the sizes ranged between 105.9 ± 15.9 and 166.8 ± 26.3 nm, and the polydispersity index values varied from 0.371 to 0.612. The preparation with the molar ratio 1:5 of HPβ-CD and DPC was selected for the ethyl acetate fraction of frankincense for further studies. Encapsulation efficiency was 87.39% ± 1.99, the size was 244 ± 17.54 nm, and the zeta potential was −25.4 ± 4.1 mV.

Studies of drug release from nanosponges evidenced a biphasic behavior, with an initial quick release lasting for the first 6 h, reaching about 67% of drug release, followed by a slow release for 24 h (up to 69%). R^2^ values were confirmed as a superior fit to Higuchi’s model if compared to the zero- and first-order kinetic models. In vivo studies with female Wistar albino rats were used after treatment with talc to induce respiratory distress. For each rat, the formulation was instilled in each nostril for 4 weeks following cessation of exposure to talc powder. For each rat, the presence of symptoms of respiratory distress, such as either apnoea or dyspnoea manifested by cyanosis around the mouth, panting or lethargy were evaluated. No signs were present in the rats treated with frankincense nanosponges. In addition, at the end of the treatment, blood sampling for biochemical assay and dissection for histopathological examination were carried out. The nanosponges loaded with frankincense significantly reduced Intercellular Adhesion Molecule 1 (ICAM-1), leukotriene B4 (LTB4) and interlukin 4 (ILβ 4), in agreement with a significant reduction in inflammatory biomarkers. Additionally, the histopathologic profiles were improved when compared to the positive control group and were similar to those of the control groups [49].

A study proposed the formulation of the EO obtained from frankincense (*B. sacra*) in chitosan nanoparticles for the treatment of breast cancer [50]. Chitosan nanoparticles loaded with frankincense EO were obtained by the ionic gelation method using 2 mg/mL chitosan in 20 mL 1% acetic acid and 1 mL of the EO. Precipitation of nanoparticles was obtained by the addition of 2 mg/mL of tripolyphosphate. Their size was 80.13 nm, the zeta potential was +39.20 mV, and encapsulation efficiency was 82.7%. The nanoparticles induced the apoptosis by up-regulation of P53, Cas-3 and 9, and exhibited selective cytotoxicity on HepG2 cells (IC_50_ was 62.94 μg/mL). In addition, nanoparticles had an anti- and pro-oxidant potential for the outside and inside of cancer cells. Finally, cell migration was inhibited (92.3%) and angiogenic factors were reduced [50].

Another study reported the encapsulation in polymeric nanoparticles of EO from frankincense (*B. sacra*) obtained by hydro distillation. The principal constituents of EO were α-pinene (61%) and d-limonene (9%). The EO was loaded in nanoparticles made of polylactide-co-glycolide (PLGA) and poly-ε-caprolactone (PCL), produced by the emulsion solvent evaporation technique. Different mixture ratios were used, namely 1:1, 2:1, 3:1, 1:2 and 1:3. The optimized nanoparticles were formulated using a 1:1 ratio of PLGA and PCL. These displayed a spherical structure with a size of 230.3 ± 3.7 nm, polydispersity of 0.13 ± 0.03, and a zeta potential of −20.36 ± 4.89 mV. Encapsulation efficiency was 80.59 ± 3.37%, and release studies displayed a controlled, sustained release of frankincense EO (83.74 ± 3.34%) over 72 h. Nanoparticles encapsulated with the EO had a lower IC_50_ value with respect to non-formulated EO. In addition, there was an improvement (at least twice) in the percentage of apoptotic and necrotic cell percentages compared to the control and free EO in the on MCF-7 cell line [51].

In a further study, the EO from frankincense (from *B. papyrifera*) was encapsulated into nanoparticles made of whey protein using spray-drying technique. Major constituents of the EO were octyl acetate (37%) and nerolidol (14.7%).

A nanocomposite film was then prepared by loading these nanoparticles into frankincense resin using a solvent casting technique. The developed nanoparticles had a spherical shape with a diameter of about 151 nm and an encapsulation efficiency of about 75.9%. In the final formulation, the film had appropriate tensile strength for imparting flexible handling. In vitro release studies evidenced that AUC0-48 values were 3389 ± 62, 2385 ± 51 and 869 ± 80 for EO encapsulated in nanoparticles, the final formulation (film) and the pure EO, respectively. These data agreed with an improved sustained release of the final formulation. Additionally, the film exhibited a relevant antimicrobial activity against all the examined microorganisms, *Pseudomonas aeruginosa* and *Micrococcus luteus*. The hemolytic activity of the film was very weak, evidenced by a very high biocompatibility. In an animal wound healing model, the therapeutic activity of the film containing only the resin or the whole film containing the nanoparticles, expressed as percentage of wound closure, were about 98.65% and 99.81%, respectively, compared to the blank group (87.48%). The superior efficacy of the film containing the nanoparticles was related to the improved collagen deposition of EO [52].

### 3.2. Myrrh

Myrrh is an aromatic oleogum resin obtained from *Commiphora* species (Burseraceae), small trees or shrubs having rough and thorny branches. The species distributed from the southern part of Arabia to the northeastern part of Africa (mainly Somalia) and the northeastern part of Kenya are responsible for the production of true myrrh. However, other *Commiphora* species that produce resins are found in Sudan, the south of Arabia, Eritrea, Kenya, Ethiopia and Somalia. Myrrh is obtained by secretory tissues found on the stem bark from incisions in the bark and dries into small clumps of sap. The species *Commiphora myrrha* (Nees) Engl. is responsible for the production of true myrrh. This species has synonyms: *Balsamodendron myrrha* Nees or *C. molmol* Engl [53].

Myrrh contains 7–17% volatile oils, 25–40% alcohol soluble resins and 57–61% water-soluble gum. Volatiles are mainly represented by 2-cyclohexen-1-one, 4-ethynyl-4-hydroxy-3,5,5-trimethyl (12%), β-elemene (8.57%) and copaene (5.50%). Furanodienes are also present [54]. Diterpenes are principally derivatives of pimarane and abietane structures. Triterpenes are largely represented by cycloartane, dammarane and oleanane moieties. Galactose, arabinose, 4–0-methyl-glucuronic acid and xylose are the major components of the water-soluble gum [55].

Myrrh has been traditionally used since time immemorial as an anti-inflammatory and wound-healing product, both in the Eastern part of the world and all the Mediterranean area. It was considered a very precious natural product, and it is mentioned in the New Testament as one of the three gifts (with gold and frankincense) that the magi “from the East” presented to the Christ Child (Matthew 2:11). Jesus was offered wine and myrrh at his crucifixion (Mark 15:23) [56].

Reported biological activities of myrrh are anti-inflammatory, antiproliferative, cardiovascular, antioxidant, anti-microbial, neuroprotective, anti-diabetic, analgesic, and anti-parasitic [57].

**Figure 3:**
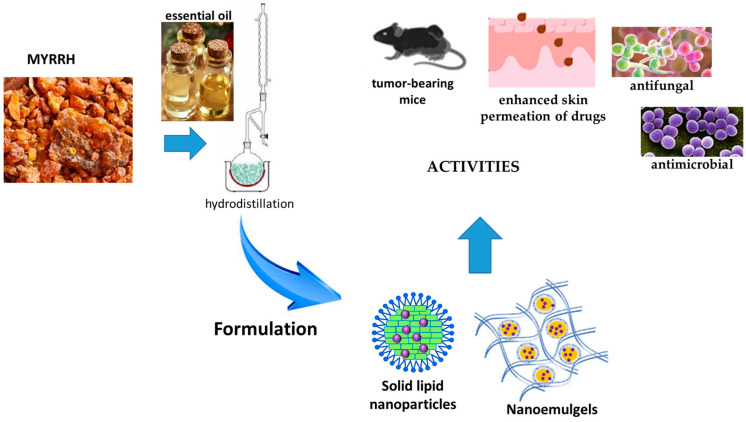
Nanovectors developed for the delivery of myrrh and main effects.

Due to its poor water solubility and high volatility, nanoencapsulation of EO for cutaneous administration has been reported. The nanocarriers developed with myrrh and the related activities are reported in Figure 3.

Solid lipid nanoparticles for the oral delivery of EO of frankincense and myrrh were successfully developed by a high-pressure homogenization method. The main constituents of the EO were octyl acetate (21.06%) and β-elemene (2.58%). The nanoparticles were obtained using Compritol 888 ATO as the solid lipid, while soybean lecithin and Tween 80 as the surfactants. The constituent ratio was 3:2:2.5:2.5:90 of Compritol 888 AT/EO/Soy-bean lecithin/Tween 80/Water, respectively. The nanoparticles had a round shape with a mean size of 113.3 ± 3.6 nm. The zeta potential was −16.8 ± 0.4 mV, while encapsulation efficiency was 80.60% ± 1.11%. The solid lipid nanoparticles were evaluated for the anti-tumor efficacy of the formulation in H22-bearing Kunming mice. Neither saline nor blank nanoparticles had any measurable effect on tumor growth, but a significant antitumor effect (*p* < 0.01) was observed for the suspension of EO, an inclusion complex of the EOs in β-CD, and the solid lipid nanoparticles loaded with the EO at a concentration of 100 mg/kg. The inhibition was calculated as 31.23%, 34.81% and 43.66%, respectively [58].

A study compared the anti-inflammatory performance of a gel of sodium carboxymethyl cellulose (CMC, 2% *w*/*w*) in water loaded with 2% *w*/*w* of curcumin and 2%*v*/*v* Tween 80, with an emulgel and a nanoemulgel. The emulgel was obtained by jelling with 2% *w*/*w* CMC the emulsion constituted by myrrh EO (10% *v*/*v*), 2% *w*/*w* curcumin, 2% *w*/*w* propylene glycol and 2% *w*/*w* ethanol in 100 mL water. The nanoemulgel had the same composition as the emulgel, but the nanosize was obtained using a homogenizer. The size of the internal phase of the emulgel was about 1.7 µ, with a polydispersity of 0.338, while the oily globules of the nanoemulgel had a size of 130 nm and polydispersity of 0.235. The release of curcumin from the gel, the emulgel and the nanoemulgel after 12 h was 72.17 ± 3.76, 51.93 ± 3.81 and 62.0 ± 3.9%, respectively. Skin permeation of curcumin was significantly (*p <* 0.05) enhanced with the application of the nanoemulgel, which had the best steady-state transdermal flux rate (108.6 ± 3.8 µg/cm^2^·h) and the greatest improvement ratio (7.1 ± 0.2). In vivo studies using a carrageenan-induced rat paw edema proved that the nanoemulgel displayed the lowest percentage of swelling (26.6% after 12 h). Finally, all the developed formulations revealed no sensitivity reaction after application on the skin of the rats’ backs [59].

A nanoemulgel based on myrrh EO was developed and loaded with fusidic acid to evaluate the antibacterial activity after skin application. The nanoemulsion formulation was prepared using different concentrations of myrrh essential oil, Tween 80 as a surfactant and Transcutol P as co-surfactant, using the experimental design. Optimization of the formulation was based on the particle size and percentage of in vitro release. The optimized formula was produced with 1.5 g of myrrh EO, 1.48 g of Tween 80 and 1.717 g of Transcutol P. Fusidic acid was added to the lipid phase, and sodium carboxymethyl cellulose was used as a jelling agent. The resulting size of the internal phase of the nanoemulsion was about 114 nm. The in vitro release of fusidic acid through a cellophane membrane from the nanoemulsion and the nanoemulgel was evaluated and compared to free fusidic acid. The latter released, after 60 min, almost 52.2% of fusidic acid in the medium, reaching 99.5% after 120 min. The released percentage of fusidic acid from the nanoemulgel and from the nanoemulsion were 80.3 ± 5.53 and 59.3 ± 5.1%, respectively, over 180 min. A kinetic study evidenced that the nanoformulations followed Higuchi modeling. The stability of both nanoformulations was assessed at 4 ± 1 °C and 25 ± 1 °C with a study protocol performed for 1 and 3 months. An ex vivo study evidenced that the steady state transdermal flux rate of fusidic acid from the suspension was 35.9 ± 4.1 µg/cm^2^·h (*p* < 0.05). On the other hand, the flux of fusidic acid from the nanoemulgel was significantly increased by 3.10 ± 0.13-fold, (111.2 ± 4.5 µg/cm^2^·h) compared to that from nanoemulsion (68.7 ± 5.0 µg/cm^2^·h), (*p* < 0.05).

No irritation was observed upon applying the nanoemulgel to animal skin. Finally, the nanoemulgel exhibited enhanced antibacterial activity against a wide variety of bacteria when compared to a conventional gel and or a marketed cream. It was very active against *Staphylococcus aureus*, *Bacillus subtilis*, *Enterococcus faecalis*, *Candida albicans*, *Shigella*, and *E. coli* (*p* < 0.05) [60].

### 3.3. Guggul

Guggul, or false myrrh, is obtained from *Commiphora mukul* (Stocks) Hook (Burseraceae) [syn. *Commiphora wightii* (Arnott.) Bhandari or *Balsamodendron mukul* (Stocks) Hook]. It is distributed in the dry regions of the Indian subcontinent—mainly India, Pakistan and Bangladesh—in the Arabian Peninsula and northeast Africa, in rocky dry areas. Traditionally, guggul is used to treat a wide variety of disorders, including atherosclerosis, hypercholesterolemia, obesity, rheumatism and allergic dermatitis. The oleogum resin is obtained from cracks and fissures of the bark, and it is a complex mixture of different compounds, including gums (32% *w*/*w*), oleogum resin (38% *w*/*w*) and EOs (1% *w*/*w*). The oleogum resin includes steroids, sterols, terpenes and aromatic compounds.

Active constituents are mainly related to Z- and E-stereoisomers of guggulsterones (4,17(20)-pregnadiene-3,16-dione derivatives), which are present in a concentration of 4.0–6.0% and are very active for the treatment of inflammation, nervous disorders, hyperlipidemia and associated cardiac disorders [61,62].

**Figure 4:**
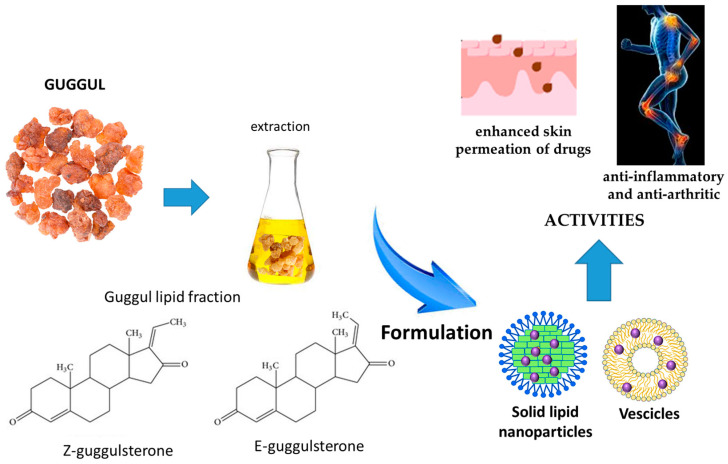
Nanovectors developed for the delivery of guggul and main effects.

Nanovectors developed with guggul, and their main effects are reported in Figure 4.

Guggul lipid, an ethyl acetate extract of guggul, containing guggulsterones, was used to formulate solid lipid nanoparticles for the transdermal delivery of diclofenac sodium salt. The nanoparticles were prepared by the melt-emulsion sonication and low-temperature solidification methods using Poloxamer 188 as the surfactant, and the biopharmaceutical properties were compared to conventional glyceryl monostearate- and stearic acid-based solid lipid nanoparticles. The percentage of the drug was 1% *w*/*w*, while guggul percentages were 2.5, 5 or 7.5% *w*/*w*. The size range of the guggul nanoparticles loaded with the drug was between 98.12 and 137.6 nm, smaller than the conventional solid lipid nanoparticles. The lowest polydispersity index value was 0.195 in nanoparticles containing the highest amount of guggul lipid (7–5% *w*/*w*). The zeta potentials were in the range of −11 to −15 mV, the lowest containing 7.5% *w*/*w* of guggul lipid. Encapsulation efficiency was between 65 and 90% (nanoparticles containing 7.5% guggul lipid). Guggul lipid nanoparticles demonstrated a more controlled and slower drug release: after 24 h, in the range 73.54–87.82%, with the slower release of those containing the higher amount of guggul lipid. The formulation with the higher amount of guggul lipid was stable for 180 days at 40 ± 2 °C and 75 ± 5% relative humidity. This formulation was incorporated into a gel using Carbopol 934 (1% *w*/*w*) to evaluate the in vivo skin drug permeation and the inhibition of skin inflammation. A very fast and high drug permeation, resulting in a maximum edema inhibition, without inducing an irritant reaction, was found. The formulation showed a 104.68 times higher drug content than a commercial gel of diclofenac sodium salt. It showed an almost 8.12 times higher C_max_ than the conventional gel at 4 h, and the AUC value was 15.28 times higher than the AUC of conventional gel. Edema inhibition was up to 69.47% already in the first hour [63].

In a further study, the employment of guggul lipid in nanovesicle formulations for transdermal delivery of aceclofenac was investigated. The nanovesicles were produced by the lipid film hydration method using guggul lipid, cholesterol and dicetyl phosphate (ratio 7:3:1 or 5:3:1 or 3:3:1) and loaded with 1% *w*/*w* aceclofenac. The performance of these vesicles was compared with that of conventional liposomes loaded with aceclofenac (1% *w*/*w*). A smaller particle size, higher homogeneity and drug entrapment efficiency, and a slower cumulative drug release were found for the vesicles containing guggul when compared with the conventional liposomes. The most promising vesicle was that containing a ratio of 7:3:1 of guggul lipid, cholesterol and dicetyl phosphate. The size was 121 ± 1.1 nm, the polydispersity index was 0.153 ± 0.004 and the zeta potential was −25 ± 1.1 mV. Encapsulation efficiency was 78.9 ± 1.1%. The formulation was appreciably stable for over 6 months at 4 °C. A gel was produced with the vesicles using Carbopol 934 (1% *w*/*w*), and there was evidence, for the vesicle formulation, of a controlled release of drug over 24 h, while the gel containing plain drug released 98.3% of the drug in only 6 h. The calculated *C* _max_ value was 7.32 μg/mL, and the *T*_max_ value was 8 h. The formulation containing guggul inhibited the edema by 90.81% in 6 h [64].

### 3.4. Mastic

Mastic (or mastic gum) is a resin obtained as a trunk exudate of the mastic tree from *Pistacia lentiscus* var. *chia* (Anacardiaceae), native through the Aegean and Mediterranean regions and extensively cultivated on the Greek island of the Aegean Sea, Chios. Mastic is a very a complex mixture of constituents and include mono-, sesqui-, and triterpenoids, as well as traces of phenolic compounds, mainly represented by simple phenolic acids such as vanillic, gallic, trans-cinnamic, o-coumaric and protocatechuic acids Triterpenes are the major chemical group (65–70%) of the total resin weight, while the polymer of mastic gum, which constitutes about 25–30% of the dry weight, is represented by trans-1,4-poly-β-myrcene polymer. The triterpenes are mainly tetracyclic and pentacyclic triterpenes, represented by derivatives of 12-oleanene, 18-oleanene, 28-nor-17-oleanene, 7-tirucallene, 24,25-dehydro-7-tirucallene, 8-tirucallene, 24,25-dehydro-8-tirucallene, dammarane, lupine, lupene and 12-lupene [65]. The EO represents about 3% or 13% of the resin according to the different harvesting methods. It is produced by hydro distillation, and major constituents are α-pinene (30–75%), myrcene (3–60%) and β-pinene (up to 6%) [66].

Since ancient times, mastic gum has been utilized for the treatment of digestive and gastric diseases. Nowadays, many studies have demonstrated interesting biological activities, including antimicrobial, antioxidant and anti-inflammatory [67]. Mastic is reported to have preventive effects of low-density lipoprotein (LDL) oxidation and as a novel wound-healing agent. Besides the demonstration of its strong activity against *Helicobacter pylori*, the principal cause of peptic ulcer, additional antimicrobial activities have been reported against different Gram-positive and Gram-negative bacteria, including *Salmonella enteritidis*, *Pseudomonas fragi*, *Lactobacillus plantarum*, *Staphylococcus aureus*, *Escherichia coli*, *Pseudomonas aeruginosa* and different *Streptococcus* species [65].

Since 2015, the Committee on Herbal Medicinal Products (HMPC) of the European Medicines Agency (EMA) has allowed its use as a traditional herbal medicinal product for mild dyspeptic disorders, minor skin inflammation and wound healing [7].

Additionally, it is widely used in food products, especially cakes, cookies, milky desserts/sweets, ice-cream, yogurts, candies and chewing gum, and also in alcohol-containing drinks (mastiha liquor, ouzo) [65].

**Figure 5:**
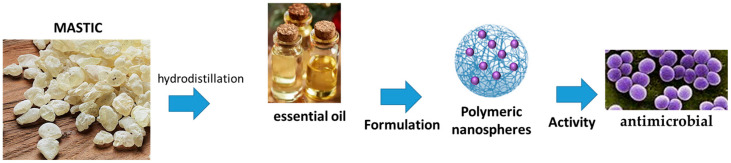
Nanovector developed for the delivery of mastic and main effects.

Besides its interesting activities, issues like its poor water solubility and great volatility of the fraction of the EO, as well as the caustic effect of mastic when applied in large quantities, the development of optimized topical nanoformulations of EOs isolated from mastic has been reported in the literature. Nanovector developed with mastic are reported in Figure 5.

A study reported the development of polymeric nanoparticles encapsulated with EO and prepared by a solvent evaporation method. The main constituents of the EO were: α-pinene (82.95%), myrcene (2.85%), β-pinene (2.06%), linalyl acetate (1.29%), trans-verbenol (1.36%), linalool (1.00%) and limonene (0.88%). Nanoparticles were developed using poly(lactic acid) (PLA) as shell material, due to its biocompatibility and biodegradability, while two surfactants, poly(vinyl alcohol) (PVA) and lecithin (LEC), were used as external polymer. Size, polydispersity index and ζ-potential between PLA/PVA and PLA/LEC nanoparticles had no significant differences (*p* > 0.05). Sizes, polydispersity and zeta-potential were 239.9 nm, 0.081, −29.1 mV, and 286.1 nm, 0.167, −34.5 mV, respectively. Encapsulation efficiency was clearly higher for PLA/PVA nanoparticles when compared with that of PLA/LEC nanoparticles (37.45% vs. 9.15%, respectively). The percentages of the EO in the formulations were 2.51 ± 0.06 and 0.59 ± 0.06, respectively. Nanoparticles made of PLA/PVA remained stable for 60 days, and the in vitro release study indicated gradual release of the EO from these nanoparticles. The Minimum Inhibitory Concentration (MIC) of the EO was determined against *Escherichia coli* and *Bacillus subtilis* using gentamicin as the reference. The gentamicin MIC was 0.25 ± 0.08 μg/mL and 0.05 ± 0.02 μg/mL, respectively. The MIC of the unformulated EO was 5.00 mg/mL for *E. coli*, while for *B. subtilis*, a more efficient inhibition of growth was found (1.25 mg/mL). No inhibitory effect of the nanoparticles was reported because after 3 h of the study, only 30% of the EO was released from the nanoparticles [68].

### 3.5. Turpentine

Turpentine, the resinous exudate obtained from coniferous trees, particularly those of the genus *Pinus* (Pinaceae family), is the term that originally referred to the whole oleoresin, but it is now commonly referred to its volatile fraction only, which is obtained by various distillation techniques, obtaining the semifluid portion of turpentine and a nonvolatile fraction called rosin. Resin is accumulated at up to 10–20% of the dry mass in stems, branches, needles and even cones of *Pinus* species and stored in specialized structures [69].

Rosin, also called colophony, mainly consists of diterpenoids (more than 70%), derivatives of abietic and pimaric acids. Turpentine is primarily constituted of monoterpenes, α-pinene (70–85%), β-pinene (11–20%) and limonene (1–7%). Other components in small amounts are camphene, car-3-ene, β-myrcene, longifolene, β-caryophyllene and caryophyllene oxide [70].

Plant species used for turpentine production include maritime pine (*Pinus pinaster*), principally distributed in France, Italy, Portugal and Spain; Aleppo pine (*P. halepensis*), distributed in Greece and Spain; longleaf pine (*P. palustris*) and slash pine (*Pinus elliottii*), distributed in USA; Masson’s pine (*P. massoniana*) from China; *P. roxburghii* (*P. longifolia*), distributed in India and Pakistan; Sumatran pine (*P. merkusii*), distributed in Malaysia; and *P. radiata* from New Zealand [71].

Coniferous plants have been used as a medicinal source for various ailments since prehistoric times. Particularly, different parts of the *Pinus* spp. (bark, leaf, cone and resin) were prescribed to treat respiratory problems, due to its diaphoretic, rubefacient, antiseptic, diuretic, stimulant and febrifuge properties. The resin was principally used in wound healing and injury, and to treat abscesses and furuncles. In the Materia Medica by Matthioli, the resin and oleum of coniferous trees were largely reported curative emplastrum for wounds and verrucas, and an adjuvant treatment of urinary complaints. *Vinum medicinalum* containing *Pinus* plant parts and resin, was among one of the best formulations reported by Matthioli [72,73].

At the beginning of the 21st century, the therapeutic interest of pine species gradually disappeared from Pharmocopoeias and medical texts, and presently, only *Pinus sylvestris* aetheroleum is officially listed in The European Pharmacopoeia [74].

In recent times, turpentine has been exploited as a potent antiseptic agent in ointments for treating several skin infections, evidencing a great therapeutic potential in clinical trials both against folliculitis and bacterial skin diseases. The anti-inflammatory, antirheumatic, diuretic, analgesic activity on bronchial secretion and pulmonary and genito-urinary tract infections are also evidenced. Finally, some studies have demonstrated possible use in the treatment of neurodegenerative disorders [75,76].

**Figure 6:**
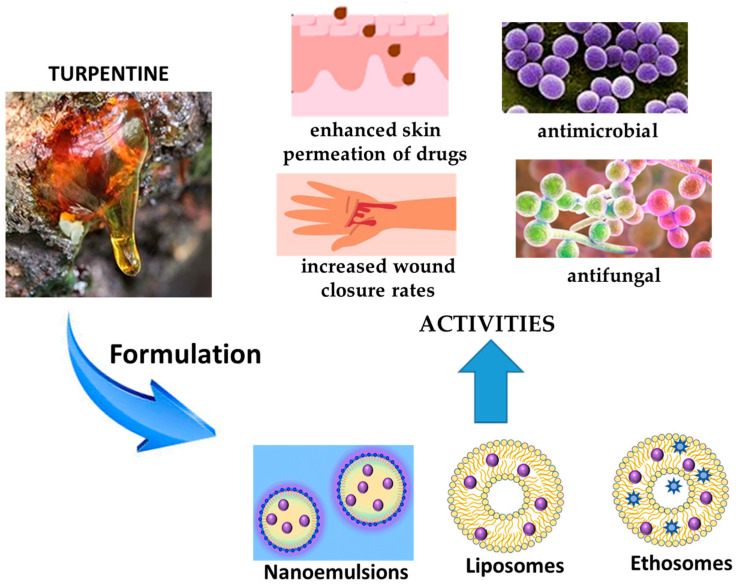
Nanovectors developed for the delivery of turpentine and main effects.

In nanotechnology, turpentine oil has been exploited as both active phytocomplex and enhancer excipient due to its capability of disrupting the stratum corneum ordered lipids (Figure 6).

This effect was evidenced by Oskuie and co-workers, who demonstrated that the presence of turpentine in liposomal and ethosomal nanoformulations increases the transdermal permeation of fluconazole. Liposomes and ethosomes were prepared by the film hydration method and hot method, respectively, with the addition of turpentine in the lipid phase at the dosage of 10, 15 or 20 mg/mL. The particle size of liposomal formulations ranged from 68.8 to 367.0 nm, with a polydispersity <0.41. Ethosomes had sizes in the range 248–340 nm, with a polydispersity < 0.36. Zeta potentials were negative for both types of formulations, from −6.10 to −7.82 for liposomes, and from −10.40 to −7.51 mV for ethosomes. The authors found that the addition of turpentine decreased the size of the two formulations, ameliorated the encapsulation efficiency (up to 90%), and increased by five-fold times the ex-vivo permeation of the antifungal drug. The results revealed that the higher permeation of the promising carriers was correlated to the increment of the vesicle fluidity. The presence of turpentine did not impair the fungicidal activity of fluconazole [77].

The encapsulation of turpentine in a nanoemulsion was attempted to hinder the biofilm formation of methicillin-resistant *Staphylococcus aureus*. The nanoemulsion was obtained via the ultrasonic emulsification method using Tween 80 as the surfactant. The oil droplet displayed a size ranging between 22.5 and 26.5 nm, depending on the concentration of the surfactant, with a polydispersity lower than 0.25. The nanoemulsion showed a similar antiradical scavenging activity to turpentine oil. Thermodynamic stability studies revealed homogeneous dispersion of the droplet sizes, confirming the stability of nanoformulation. The antibacterial and antibiofilm activities of turpentine nanoemulsion were higher than those of raw turpentine. Nanoformulation enhanced by two-fold the antagonistic activity against *S. aureus* in comparison with turpentine, with a MIC of 0.039% (*v*/*v*). The percentage of biofilm disruption was around 70.83% [78].

In the Nordic countries, especially in Finnish Lapland, the resin of another Pinaceae, *Picea abies*, is used. The genus name *Picea* is derived from the Latin name *Pix picis*, which means pitch. The common name of this resin is Norway spruce, used for centuries to treat skin diseases, including acutely and chronically infected wounds, sores, ulcers and abscesses. The constituents of this resin are similar to those of other coniferous resins, mainly represented by resin acids, including abietic, neoabietic, dehydroabietic, pimaric and isopimaric acids. Some hydroxycinnamic acids, acids and lignans such as pinoresinol, lariciresinol, and matairesinol are also present. The ancient formulation is prepared by boiling resin with butter or other animal fat [79,80].

Vater and co-workers investigated the wound-healing effect of the encapsulated Norway spruce into lecithin-based nanoemulsions. Different O/W nanoemulsions made of lecithin Lipoid^®^ S 75 (5% *w*/*w*) and jojoba or sunflower seed oil (10% *w*/*w*) or synthetic medium-chain triglycerides (10% *w*/*w*) were produced via high-pressure homogenization. Spruce was loaded at 0.1% *w*/*w*. Mean droplet sizes of nanoemulsions containing the natural oils were about 230, with a polydispersity index of about 0.23 and a zeta potential of about −68 mV. The nanoemulsion obtained with the synthetic triglycerides had a size of 168 nm, polydispersity of 0.19 and zeta potential of −76 mV. The MTT test evidenced a high skin cell viability at concentrations 1:400 when compared with the control. Moreover, in scratch assays, the nanoemulsions with spruce balm showed significantly increased wound closure rates compared to both negative and positive controls. Particularly, in primary human fibroblasts, smaller cell-free gaps after 48 h were found for the nanoemulsion loaded with spruce, reaching, in general, a wound closure of 73–75% [81].

### 3.6. Galbanum and Asafoetida

Galbanum and asfoetida are resins produced by plants of the *Ferula* genus (Apiaceae), which includes more than 170 species distributed in central Asia, Mediterranean countries and northern Africa. Among these, *Ferula assa-foetida* L. and *Ferula gummosa* Boiss. are the most important species, producing the oleogum resins asafetida and galbanum, respectively, which are extracted from the roots and stems of the plants after incision. These species are diffused in Iran [82]. Remarkably, galbanum, when cooked, releases a particularly appreciated garlic-like smell, and it is reported in the Belfrit list of botanicals that it can be used as an ingredient of food supplements. Galbanum is reported in ancient writings because it represents a sacred substance, known also as green incense, starting from the ancient Egyptians. Known as a medicinal product since antiquity, it was often employed in medicine by Hippocrates and Pliny; it has been traditionally used as carminative, laxative, antispasmodic, expectorant and sedative. According to Tabernaemontanus (1687), the oil obtained from galbanum can be applied to the neck or drunk for improving memory, hearing and sense of smell [83]. Galbanum and asafetida have a disagreeable and peculiar bitter taste. These oleogum resins are composed of a volatile fraction represented by monoterpenes (10–17%, *w*/*w*). The principal constituents are the stereoisomers E and Z of 1-propenyl sec-butyl disulfide for asafetida, and α- and β-pinene for galbanum. Other constituents are limonene, cadinene, 3-carene and ocimene. Gum represents 25% *w*/*w* and resin from 40 to 64% *w*/*w*. Principal constituents of the fixed resinous fraction are coumarin derivatives and polyphenols (phenolic acids and flavonoids) [84,85].

Several biological activities of galbanum and asafoetida are reported, specifically, cardioprotective, anticonvulsant, spasmolytic and antiepileptic effects, in addition to antibacterial and antioxidant properties. Concerning skin disorders, the oleogum resin of *Ferula* demonstrated anti-aging effects by revitalizing senescent fibroblasts and protection against UV radiations. However, no studies have aimed to develop formulations of the essential oil or the oleogum resin for such purposes [86,87].

**Figure 7:**
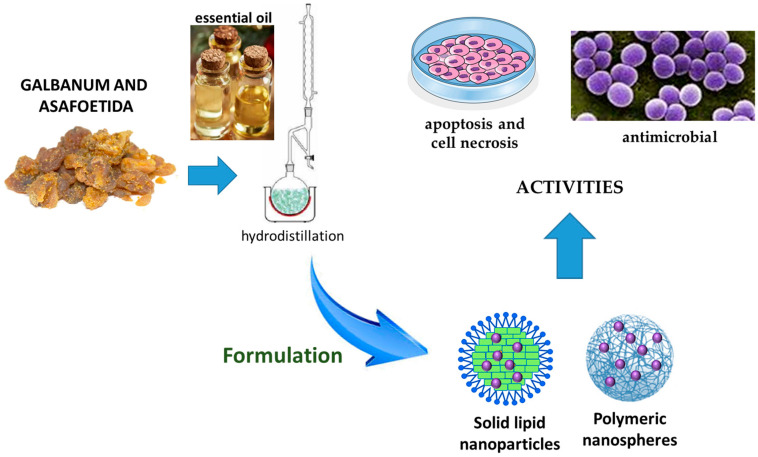
Nanovectors developed for the delivery of galbanum and asafoetida and main effects.

In the literature, nanodelivery systems were developed to increase the activity of the EOs from resins. Most of the studies focused on the incorporation of the active compounds in solid lipid nanoparticles and polysaccharide-based nanoparticles (Figure 7).

A study reported the development of solid lipid nanoparticles consisting of the EO obtained from *F. assa-foetida* seeds (100 µL), stearic acid (400 mg) and lecithin (200 mg). The EO contains (Z)-sec-butylpro-penyl disulfide, (E)-sec-Butylpropenyl disulfide and Trifluoromethyl t-butyl disulfide as major constituents. The size of the nanoparticle was about 195 nm, with a polydispersity of 0.31 and a zeta potential of −32.08 mV. Nanoparticles were evaluated for their tumor-suppressive potential on NT-2 human cancer stem cells. The IC_50_ concentration of the nanoparticles was found to be 115.4 µg/mL, during 48 h incubation time, while the IC_50_ in HFF fibroblast cells was 1539 µg/mL, indicating a high selectivity for tumor cells. The study evidenced how nanoparticles can induce apoptotic death by up-regulating the expression of TNF-α, P21 and Cas3 genes, and can surprisingly suppress angiogenesis in cell-assembled extracellular matrix tissue by reducing the length and number of blood vessels [88].

EO obtained from galbanum was loaded in chitosan nanocomposites and proposed against multidrug-resistant pathogens for biomedical purposes. The monoterpene hydrocarbons α-pinene, β-pinene and β-phellandrene represented the main constituents of the EO. Nanocomposites were obtained by the emulsification method, and the ratio between chitosan and the EO was 0.2. Nanoparticles had a spherical shape, and the loading of EO decreased the size from about 480 nm to 250 nm; loading values were in the range between 0.3 and 0.5 g. The antibacterial activity of nanoparticles against strains of *Escherichia coli*, *Staphylococcus aureus*, *Pseudomonas aeruginosa*, *Salmonella typhimurium* and *Bacillus cereus* were evaluated with the broth micro-dilution method. A nanocomposite concentration of 0.39 μL/mL stopped the growth of pathogens, while the concentration of 1.4 μL/mL was able to kill the bacteria. The study evidenced that Gram-positive bacteria were as sensitive as Gram-negative ones [89].

### 3.7. Dragon’s Blood

Dragon’s blood is a deep red resin, which has been used as a famous traditional medicine since ancient times by many cultures. Dragon’s blood is a non-specific name for red resinous exudations from quite different plant species endemic to various regions around the globe that belong to the genera *Dracaena* (family Asparagaceae, Africa) and *Daemonorops* (family Arecaceae, South-East Asia), more rarely also to the genera *Pterocarpus* (family Fabaceae, South America) and *Croton* (family Euphorbiaceae, South America). *Dracaena draco* L. is the best known; it is endemic to the Canary Islands and Morocco. The chemical composition of the red resin is species-specific. The main active compounds belong to the classes of alcohol esters, phenolic acids, tannins, proanthocyanidins, steroids, alkaloids, flavonoids and terpenes.

Dragon’s blood has an astringent effect, and it has been traditionally used as a hemostatic and antidiarrheic drug. Some of the early Greek and Roman writers, among them Dioscorides and Pliny, reported high esteem for the medicinal properties of dragon’s blood (from *D. draco*). The genus name Dracaena is derived from the Greek word “drakainia”, meaning a female dragon. The resin of *D. draco* is exuded from the wounded trunk or branches of the tree. *D. cochinchinensis* (Lour.) S.C. Chen is another species used in China as source of Dragon’s blood, characterized by the presence of Loureirin A and B, two dihydrochalcones.

**Figure 8:**
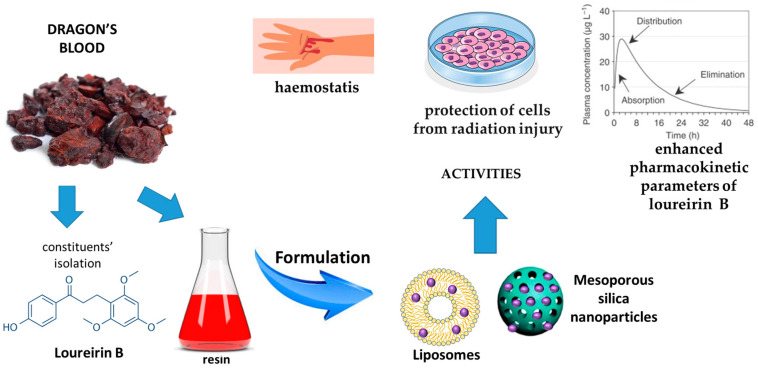
Nanovectors developed for the delivery of dragon’s blood and main effects.

In European medicine, dragon’s blood was formerly used in dysentery and diarrhea and as an astringent in tooth powders [90]. Nanocarriers developed for dragon’s blood and their effects are reported in Figure 8.

The antiradical activity of loureirin A and B from *D. cochinchinensis* and their capability of protecting the skin against UV rays, preventing skin aging and tumors, and with a central role in wound healing and skin rejuvenation by increasing collagen synthesis, have been reported. Additionally, these constituents have a potent antimicrobial effects in wound healing, preventing and treating hypertrophic scars [91,92].

Loureirin B is the most interesting constituent, but the limited pharmacokinetic profile due to its hydrophobic character restricts its clinical uses. For this reason, nanoliposomes made of phosphatidylcholine (in the range 150–300 mg/mL), cholesterol (in the range 50–150 mg/mL), loureirin (in the range 10–30 µg/mL) and Tween 80 (in the range 2–6%) were developed using the thin-film evaporation technique. All the formulations were vesicles with particle sizes in the ranges from 58 to 94 nm. Drug-loading capacity enhanced from 58 to 94% upon raising lipid concentration from 150 to 250 mg/mL. The vesicle composed of phosphatidylcholine (300 mg/mL), cholesterol (100 mg/mL), loureirin (30 µg/mL) and Tween 80 (6%) represented the best formulation. It displayed drug entrapment efficiency of 74.5%, a diameter of 93.62 ± 0.73 nm, polydispersity of 0.29 ± 0.01 and zeta potential of −51.2 ± 2.04 mV. In vitro flow cytometry studies evidenced that the optimized vesicles restored the radiation injury in viable cells from 79.4 to 89.9%, early apoptosis from 3.5 to 0.2%, necrosis from 14.8 to 9.8%, and late apoptosis from 2.3 to 0.0%. In vivo studies evidenced an enhanced pharmacokinetic parameters, including maximum concentration of loureirin B, elimination rate half-life, area under the curve and plasma clearance to 3.247 ± 0.631 ng/mL h, 14.765 ± 10.780 min, 2.957 ± 0.201 ng/mL h and 0.132 ± 0.901 ng/mL, respectively [93].

Dragon’s blood from *D. cochinchinensis* was loaded in mesoporous silica nanoparticles, and their performance for rapid haemostasis and antibacterial activity were evaluated. The mean particle size of nanoparticles was around 50 nm and the dimension of the pores was about 4 nm. Dragon’s blood was added at the weight ratios of 5%, 10% and 20%. The loading abilities using a mass ratio were 4.57%, 8.76% and 16.68%, respectively. In vitro studies evidenced that the release of pure dragon’s blood in solution was modest (at 120 min, it was not more than 2%). Conversely, the performance of the formulations with increasing amounts of dragon’s blood reached equilibrium after 30 min, with cumulative releases of 97.60%, 98.37% and 98.32%, respectively. The clotting times of the three formulations were 108.33 ± 6.23 s, 89.67 ± 2.05 s and 107.33 ± 6.55 s, respectively, quicker than those observed for the empty nanoparticles (151.33 ± 7.41 s) and the unformulated dragon’s blood (220.33 ± 25.95 s). The optimal haemostatic efficiency was the formulation containing 8.76% dragon’s blood. Finally, from the in vivo studies, it was found that the haemostasis time was reduced after application of the nanoparticles or if not formulated with dragon’s blood, but the best performance was obtained with the three formulations containing 4.57%, 8.76% and 16.68% dragon’s blood. Reduction was 121.00 ± 8.00 s, 81.00 ± 5.57 s, and 86.67 ± 1.53 s, respectively. The blood loss of nanoparticles containing 4.75% dragon’s blood (0.754 ± 0.058 g) was greater than the formulations containing 8.76% and 16.68% dragon’s blood [94].

### 3.8. Copaiba

Copaiba is a liquid oleoresin obtained from the tree by punching a hole into the trunk, collected from several trees of plants belonging to the genus *Copaifera* (Fabaceae family). The genus *Copaifera* is distributed worldwide in tropical regions of Africa, Central America and South America; the largest number (28) of *Copaifera* species are from Brazil, sixteen of them native of Brazil, mainly from the Cerrado and Amazon regions. The most important commercial sources of copaiba are *C. langsdorffii* Desf., *C. officinalis* (Jacq.) L., and *C. reticulate* Ducke. The EO fraction of copaiba represents 33% in *C. langsdorffii*, 87% in *C. officinalis* (Jacq.) L. and 68% in *C. reticulata*. The sesquiterpenes β-caryophyllene, β-caryophyllene oxide, trans-α-bergamotene, and β-bisabolene are sesquiterpenes present in copaiba. More representative diterpenes are clerodane, kaurane and labdane derivatives, mainly copalic acid, kaurenoic acid and α-copaene. Copaiba from different sources has shown antibacterial, anti-inflammatory, antileishmanial, antiproliferative, antitrypanosomal and wound-healing activities [95].

Copaiba is very active against Gram-positive species (*Staphylococcus aureus*, methicillin-resistant *S. aureus*, *Staphylococcus epidermidis*, *Bacillus subtilis*, and *Enterococcus faecalis*), with minimum inhibitory concentrations ranging from 31.3–62.5 µg/mL. Minor activity was found against dermatophyte fungi (*Trichophyton rubrum* and *Microsporum canis*), while copaiba was not active against Gram-negative bacteria and yeast [96].

The medicinal use of copaiba was first described in the 16th century for the treatment of wounds. Its use decreased in the 18th century, but nowadays, there is an increasing interest due to potential health benefits, especially as wound healing for the skin and oral cavity, antibacterial for treating acne, and antiparasitic against cutaneous leishmaniasis. Finally, due to the high amount of EO, copaiba represents a skin penetration enhancer [97,98].

**Figure 9:**
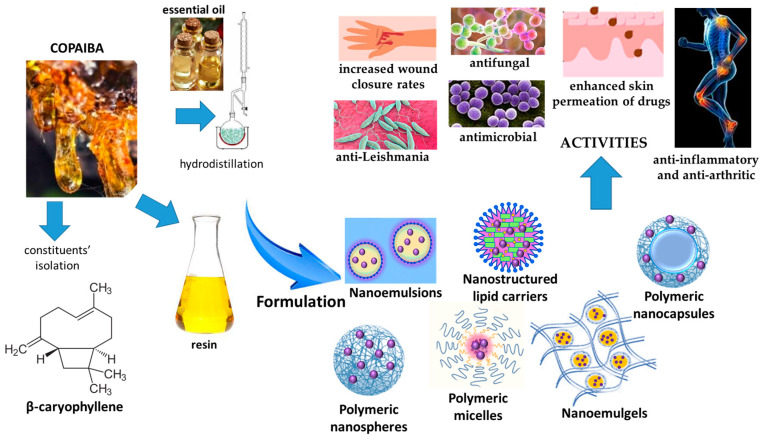
Nanovectors developed for the delivery of copaiba and main effects.

Copaiba has been employed for the production of nanocarriers both as a functional excipient and active phytocomplex to enhance its activity. The successful incorporation of the oleoresin was achieved by developing nanoemulsion, polymeric micelles, and lipid- and polymeric-based nanoparticles (Figure 9).

Concerning nanoemulsion dosage forms, the impact of the formulation process, the surface charge, and the embedding into hydrogel on the efficacy of the resin in treating edema and skin inflammation were investigated in depth. In the first study, Dias and coworkers optimized the formulation of the copaiba-based nanoemulsion with the resin from *Copaifera multijuga* Hayne. The 2_IV_^4−1^ fractional factorial design was used for the development of the formulation, and it indicated that the most adequate composition is copaiba, medium chain triglycerides (MCT), Span 80 at high level and Tween 20 at low level. Two different preparation methods, variable concentrations and diverse ratios of surfactants were investigated to obtain a stable colloidal system with a high content of β-caryophyllene, the main volatile compound of the resin (about 30% *w*/*w*). The optimized nanoemulsion (copaiba 20%, MCT 10%, Span 80 3% and Tween 20 1%, *w*/*w*), developed by the high-pressure homogenization technique, exhibited a particle size of 250 nm, a negative zeta potential (−31 mV) and excellent homogeneity (polydispersity < 0.1). Encapsulation evaluated as β-caryophyllene was around 100%. The formulation showed good stability since the phytochemical properties, and the sesquiterpene content did not change over 30 days when stored at 4 °C [99].

In a further study, the skin permeation of β-caryophyllene and the antiedematogenic effect of the optimized negatively-charged formulation was compared to a positively-charged nanoemulsion obtained using cetyltrimethylammonium bromide as the surfactant. The selected nanoemulsion showed a particle size of about 200 nm, a PDI of around 0.1, and a zeta potential of 20 mV. The permeation study highlighted that the positive charge of the nanoemulsion allowed the penetration of β-caryophyllene up to the receptor fluid and increased the concentration of the sesquiterpene in the epidermis three-fold compared to the negatively charged dosage form. Conversely, the nanoemulsion charge did not affect the concentration of β-caryophyllene in the dermis. The study on mouse ear edema demonstrated that only the negatively charged nanoemulsion increased the activity of raw copaiba oil resin, which was comparable to that of the positive control (ketoprofen). Conversely, both nanoformulations presented the same antiedematogenic profile, statistically equal to ketoprofen on rat paw edema [100].

Finally, the same authors attempted to ameliorate the dosage form by embedding the optimized nanoemulsions, positively and negatively charged, into a polymeric hydrogel, aiming at increasing the viscosity of the formulation to have a practical application. Three polymers were tested: Carbopol, hydroxyethylcellulose and chitosan, but only Carbopol and hydroxyethylcellulose hydrogels did not interfere with the nanoemulsion droplet size and polydispersity index. The hydrogel formed by hydroxymethylcellulose showed the highest compatibility with the nanoemulsions and remained stable for 1 year. In a skin permeation assay, both formulations loaded with negatively and positively charged nanoemulsions showed high retention in the epidermis (9.76 ± 2.65 μg/cm^2^ and 7.91 ± 2.46 μg/cm^2^, respectively), while the retention in the dermis was very low (2.43 ± 0.91 and 1.95 ± 0.56 μg/cm^2^, respectively). They also presented permeation to the receptor fluid (0.67 ± 0.22 and 1.80 ± 0.85 μg/cm^2^, respectively). The anti-inflammatory activity was similar with edema inhibitions of 69 and 67% in mouse ear edema, while it resulted in 32 and 72% in rat paw edema, respectively. A histological study evidenced the reduction of inflammatory factors, confirming the anti-inflammatory effect from both copaiba nanoemulsions incorporated in hydrogel [101].

A further nanoemulsion was developed to test the effects of copaiba (from *C. langsdorffii*) and the EO fraction against strains from the *Staphylococcus*, *Pseudomonas* and *Candida* genera, which are responsible for cutaneous infections. Tween 20 and Span 80 at concentrations of 1.56% and 0.44%, respectively, were employed for the formulation of nanoemulsions containing 5% of the resin or the EO extracted by hydrodistillation.

The nanoemulsions displayed a droplet size of about 200 nm, with polydispersity lower than 0.3. Overall, nanoemulsions containing the EO were more active than the copaiba whole-resin nanoemulsions, both in inhibiting microbial growth and biofilm formation also on *Candida* strains resistant to azole antifungals. Conversely, strains of *C. albicans*, *C. tropicalis* and *C. parapsilosis* were not sensitive to the EO. The bioautography performed on MIC assay demonstrated that the antimicrobial activity was ascribed to the volatile terpenes α-curcumene, α-himachalene, isothujol and α-fenchene [102].

Finally, nanoemulsions were designed and developed to treat cutaneous *Leishmania infantum* and *L. amazonensis*. Copaiba (10% *w*/*v*) nanoemulsion was obtained via ultrasonication using Tween 80 and Span 80, both at 4% *w*/*v*, and displayed a unimodal population (polydispersity was 0.14) with a droplet size of 76 nm and a zeta potential of −2.54 mV. The formulation remained stable for 90 days. A toxic activity of nanoemulsion against promastigotes of both *Leishmania* species was found, inducing oval cell shape and retracted flagella. In the macrophage cultures, a reduction of *L. infantum* and *L. amazonensis* infection levels was evidenced. Nanoemulsion significantly reduced the cutaneous lesions induced by *Leishmania* species using BALB/c mice as a model, even though a complete cure was not noticed. No comparison of the activity with raw oleoresin was reported [103].

A micellar dispersion of copaiba (from *C. langsdorffii*) was obtained with pluronic F-127 and tested for antifungal formulation against fungi of the genus *Paracoccidioides*. Copaiba concentration was 2 mg/mL, and Pluronic F-127 was used at the concentration of 2.4%. The micelles’ average size was 34.97 nm, with a polydispersity of 0.12. After lyophilization and reconstitution in PBS (pH 7.4), polydispersity was 0.3, with the prevailing population (85%) having a mean diameter of 29.98 nm, while the smallest population (15%) had a diameter in the range between 200 and 1200 nm. The formulation had a promising antifungal activity against *Paracoccidioides*, even if there was lower fungicidal activity than with the pure resin, probably due to the interaction of the hydrophilic chains of Pluronic F-127 with the fungal membrane, which hindered the access of the oil to the cell wall of the microorganisms. No cytotoxicity or hemolytic effect was observed at the minimum inhibitory concentration (MIC) of both the resin and the formulation. When the resin and the formulations were combined with amphotericin B, an additive effect with a reduction in MIC values was observed [104].

Polymeric nanocapsules were developed using the EO of copaiba from *C. officinalis*. The nanocarrier was prepared by nanoprecipitation using poly-ε-caprolactone (90 mg), with Span 80 (56.25 mg) and Tween 80 (112.5 mg) as surfactants, and 375 µL of EO. The nanocapsules showed a mean size lower than 250 nm, a polydispersity value around 0.2, a negative charge, and an encapsulation efficiency of about 74%, calculated as β-caryophyllene, which represented a main constituent. Nanoparticles were then embedded into a chitosan film (2% of chitosan) via casting method. The chitosan film embedding the nanocapsules demonstrated superior mechanical characteristics compared to the film obtained by incorporating the EO from copaiba in terms of thickness, tensile strength and elasticity. Both chitosan films containing 1% *v*/*v* of the EO and the film containing the nanocapsules encapsulated with the EO had pronounced antibacterial properties against *Staphylococcus aureus* and *Pseudomonas aeruginosa* [105].

Nanoparticles of copaiba (*C. officinalis*) were obtained by supercritical fluid extraction of emulsions, using a modified starch, Hi-Cap 100, as core material. First, the nanoemulsion was produced as an oil-in-water formulation (200 mL) using 1.2 g of copaiba in ethyl acetate (30 mL) and 3.4 g of Hi-Cap 100 in water (170 mL). The droplet diameter of the nanoemulsion was 261.7 ± 2.2 nm. The nanoparticles were obtained by extraction of the ethyl acetate using supercritical CO_2_. At the end of the process, the recovery of β-caryophyllene, which represents the main constituents of copaiba, was 7.3%. Finally, the suspensions were dried to obtain powder particles having a coaxial dimension of 127–178 nm [106].

Nanoparticles of poly(lactic-co-glycolic) acid containing copaiba were developed using the design of the experiments, which demonstrated that higher amounts of copaiba combined with higher amounts of surfactant (Pluronic F68) improved the encapsulation efficiency, optimized the size distribution and enhanced stability after freeze-drying. The encapsulation efficiency of copaiba was about 87%, the mean nanoparticle size with spherical shape was about 214 nm, the polydispersity index was 0.22 and the zeta potential was −13.21 mV. After 48 h of incubation with endometrial stromal cells, the nanoparticles evidenced a reduction of viability from the ectopic endometrium of patients with endometriosis and from eutopic endometriotic lesions [107].

Copaiba nanocapsules were obtained using polycaprolactone (PCL; molecular weight: 80,000) and produced using the nanoprecipitation method. The aqueous phase (53 mL) was Tween 80 (77 mg) solution. The organic phase was in acetone (27 mL), and included copaiba (160 mg), PCL (100 mg) and Span 60 (38 mg). The mean particle size of the nanocapsules was 215 nm, the polydispersity index 0.15, and the zeta potential −18 mV. These parameters were stable during over 30 days at 25 ± 2 °C. The developed nanocapsules did not induced hemolysis in erythrocytes, and they were not cytotoxic and genotoxic in lung cells at concentrations from 50 to 200 μg·mL^−1^ [108].

Nanostructured lipid carriers embedded into an emulgel were developed to improve the efficacy and the administration of copaiba from *C. langsdorffii*. The nanoparticles were produced by the hot homogenization technique using Illipe butter (200 mg) as the solid lipid and copaiba (1% *w*/*w*) and Pluronic F68 (0.5% *w*/*v*) as the surfactant. The nanocarriers exhibited a narrow size profile, around 200 nm (polydispersity < 0.14), and a negative zeta potential (about −17 mV). The emulgel was prepared using Sepineo P600 (3% *w*/*w*), propylene glycol (5% *w*/*w*), Labrafac lipophile WL 1349 (10% *w*/*w*), methyl dibromo glutaronitrile/phenoxyethanol (0.1% *w*/*w*) and water. The nanostructured lipid carrier dispersion (30% *w*/*w*) was then added to the emulgel, which had a non-Newtonian nature with pseudoplastic performance. The emulgel formulation was very effective in a skin wound model of rats and more effective in a conventional cream containing 1% copaiba [109].

Nanostructured lipid carriers were also developed in association with the drug allantoin to evaluate the efficacy against multidrug-resistant *Candida parapsilosis*. The lipid phase included cetyl palmitate (12 g), copaiba (6 g), Span 80 (2 g) and butyl hydroxy-toluene (0.1 g), while Tween 80 (4 g) and allantoin (2 g) were added to the aqueous system. The nanosystems had diameters below 120 nm, with a polydispersity of 0.125 and negative zeta potential (−10.8 mV). An unspecific fungistatic activity was evidenced for the nanoparticles, with 125 μg/mL and 7.8 μg/mL for nanoparticles loaded only with copaiba and those co-loaded with copaiba and allantoin, respectively [110].

In a further study, nanostructured lipid carriers were co-encapsulated with imiquimod (approved drug for the treatment of basal cell carcinoma) and copaiba. Two formulations were prepared. The lipidic phase of the first formulation contained poly (ɛ-caprolactone) (PCL, 0.2502 g), sorbitan monostearate (0.0962 g), copaiba (835 μL) and imiquimod (0.025 g) dissolved in a mixture of acetone (60 mL) and ethanol (7.5 mL). The aqueous phase contained Tween 80 (0.1923 g). Additionally, nanostructured carriers based on Brazilian lipids were prepared using cupuaçu seed butter (13.4 g), copaiba oil (6.6 g), imiquimod (0.2 g) and sorbitan monooleate (3.0 g) as the oil phase. The aqueous phase was prepared by mixing polysorbate 80 (3.08 g) in water. The two types of nanocarriers had similar viscosity, with the same high encapsulation efficiency values, 97.9% and 99.9% for nanoparticles made of PCL and cupuaçu seed butter, respectively. The mean sizes of the nanoparticles were 206 nm and 177 nm, respectively, while the polydispersity was 0.159 and 0.084, respectively. The zeta potential was about −12 mV for both, while the drug content was 6 and 1 mg mL^−1^, respectively. Additionally, both formulations displayed a controlled release of imiquimod, and the slowest drug release (*p* < 0.05), was found in the formulation containing cupuaçu seed butter. The in vitro performance of both nanocarriers was carried out with keratinocytes (HaCaT), evidencing a high biocompatibility of the investigated nanocarriers. An ex vivo skin permeation/penetration study was carried out using pig skin, evidencing enhanced drug retention in the skin layers and reduced drug amount in the receiver solution in the formulation containing PCL [111].

## 4. Discussion and Future Perspectives

Nanovectors are drug delivery systems of increasing importance for health products and the pharmaceutical industry because of the numerous advantages over conventional delivery platforms, improving the stability, efficacy and bioavailability of many drugs. Current research on resins has evidenced many potentialities of these natural products as therapeutic agents, evidencing the multiple cellular interactions and a great variety of different active constituents with a plethora of properties. However, the main limits of resins and their constituents are low water solubility, poor stability and bioavailability.

According to the studies reported in this review, whole resins, their fractions, EOs obtained by hydrodistillation and isolated constituents have been tested for their activity and encapsulated in diverse nanoparticles to overcome the limitations listed above. Nanosized drug delivery systems reported in the studies are mainly polymeric and lipid nanoparticles, vesicles, nanosized emulsions and inorganic mesoporous nanosystems. The most investigated resins are frankincense and copaiba, which also represent the resins with the largest use in conventional formulations, while the studies related to the other resins are very limited. It is noteworthy that nanocarriers loaded with single constituents of frankincense, 11-keto-β-boswellic acid (KBA) and 3-*O*-acetyl-11-keto-β-boswellic acid (AKBA) have demonstrated an increased bioavailability and efficacy as anti-inflammatory and antiarthritic remedies, but have also evidenced unexpected activities in vivo, such as a reduced cerebral infarct volume and improved neuronal function deficit, in animals with the middle cerebral artery occlusion, opening new horizons in the treatment of this disease. In general, the use of formulated EO led to the increased stability of the EO constituents, an improved and prolonged release of the EO and enhanced antimicrobial and antifungal activities. Concerning the wound healing studies, a very high biocompatibility, together with a strong antimicrobial efficacy of EOs and of the extreme capacity to increase wound closure rates of non-volatile terpenoid constituents, impart to resin powerful wound healing effects. Furthermore, among others, EOs from myrrh loaded in a nanoemulgel has enhanced the skin permeation of curcumin to reach an optimal steady-state transdermal flux, nanovesicles loaded with gugglusterones have optimized the transdermal delivery of aceclofenac, and copaiba loaded in nanostructured carriers improved the biopharmaceutical properties of imiquimod loaded in the same particles by optimizing the controlled release of the drug and enhancing the drug retention in the skin.

Briefly, the results evidenced from the studies reported in this review were that the developed nanoparticles were able to entrap high amounts of resins or their components, modify the release properties, enhance their cellular uptake and penetration across biological barriers and optimize the biopharmaceutical properties. On the other hand, the resins or some fractions were demonstrated to be useful in optimizing the architecture and properties of nanocarriers in their capacity to circumvent biological barriers, some components of the resins having enhanced penetration properties, opening new scenarios in the exploitation of nanocarriers to improve therapy. Although no clinical studies have been reported until now, resin-based nanovectors represent a huge platform for upgrading therapies and emerging new treatments; among others, wound healing therapy can especially benefit from the application of resin-based nanovectors due to their superiority when compared with conventional drug delivery systems, opening new clinical opportunities.

Despite these positive features of developed nanovectors loaded with resins or fractions, the main challenges for their therapeutic applications are represented by the complexity of these natural compounds, generally phytocomplexes including lipophilic and volatile constituents (EOs) plus nonvolatile lipophilic constituents (sterols, terpenoids and diterpenoids), and in many cases, also including a fraction of hydrophilic polymers highly soluble in water, which makes their standardization very difficult. Additionally, the high variability in the phytochemical profiles of resins obtained from the same species but originating from different areas, or resins from different species, make the standardization process very challenging. Fractions or single constituents could have prospective therapeutic uses for diseases which have traditional long-term treatments, principally anti-inflammatory and antirheumatic after oral or dermatological administration, and antibacterial, antifungal and to wound healing after local administration. Concerning the translation of the reported studies to the clinic, it should not be very difficult because the low toxicity of resins, from long-term traditional use, is well known. In addition, the proposed nanoformulations are completely biocompatible, and most of them are biodegradable. The excipients are largely recognized as safe, as well as the nanoformulations, as stated by the in vivo studies. Finally, the possible use of resins or their fractions as nanocarriers forming constituents open an innovative approach in which the resin fractions, such as guggul lipid (made of gugglusterones) or the EO, act as functional excipients which can increase stability skin permeability or efficacy, while decreasing undesired side-effects.
